# 3,3,6,6-Tetra­methyl-9-phenyl-3,4,5,6-tetra­hydro-9*H*-xanthene-1,8(2*H*,7*H*)-dione

**DOI:** 10.1107/S1600536809010526

**Published:** 2009-03-28

**Authors:** B. Palakshi Reddy, V. Vijayakumar, T. Narasimhamurthy, J. Suresh, P. L. Nilantha Lakshman

**Affiliations:** a Organic Chemistry Division, School of Science and Humanities, VIT University, Vellore 632 014, India; b Materials Research Centre, Indian Institute of Science, Bangalore 560 012, India; c Department of Physics, The Madura College, Madurai 625 011, India; d Department of Food Science and Technology, Faculty of Agriculture, University of Ruhuna, Mapalana, Kamburupitiya 81100, Sri Lanka

## Abstract

In the title compound, C_23_H_26_O_3_, the three six-membered rings of the xanthene system are non-planar, having total puckering amplitudes, *Q*_T_, of 0.443 (2), 0.202 (2) and 0.449 (2) Å. The central ring adopts a boat conformation and the outer rings adopt sofa conformations. The crystal structure is stabilized by van der Waals inter­actions.

## Related literature

For the biological and pharmaceutical properties of xanthenes, see: Hideo (1981 ▶); Lambert *et al.* (1997 ▶); Poupelin *et al.* (1978 ▶). For puckering parameters, see: Cremer & Pople (1975 ▶).
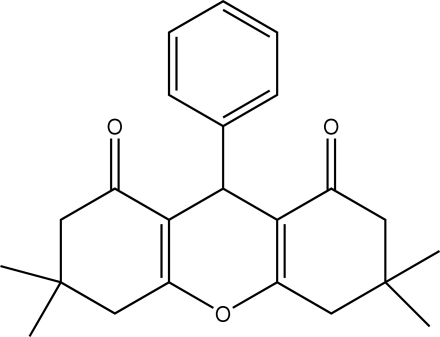


## Experimental

### 

#### Crystal data

C_23_H_26_O_3_
*M* *_r_* = 350.44
Monoclinic, 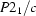

*a* = 6.0562 (5) Å
*b* = 19.7680 (18) Å
*c* = 16.4325 (13) Å
β = 97.924 (3)°
*V* = 1948.5 (3) Å^3^
*Z* = 4
Mo *K*α radiation
μ = 0.08 mm^−1^
*T* = 293 K
0.17 × 0.15 × 0.11 mm

#### Data collection

Bruker SMART APEX CCD diffractometer
Absorption correction: multi-scan (*SADABS*; Bruker, 1998 ▶) *T*_min_ = 0.987, *T*_max_ = 0.992
11861 measured reflections
4284 independent reflections
2825 reflections with *I* > 2σ(*I*)
*R*_int_ = 0.035

#### Refinement

*R*[*F* ^2^ > 2σ(*F* ^2^)] = 0.050
*wR*(*F* ^2^) = 0.138
*S* = 1.03
4284 reflections
239 parameters
H-atom parameters constrained
Δρ_max_ = 0.17 e Å^−3^
Δρ_min_ = −0.20 e Å^−3^

### 

Data collection: *SMART* (Bruker, 2001 ▶); cell refinement: *SAINT* (Bruker, 2001 ▶); data reduction: *SAINT*; program(s) used to solve structure: *SHELXS97* (Sheldrick, 2008 ▶); program(s) used to refine structure: *SHELXL97* (Sheldrick, 2008 ▶); molecular graphics: *PLATON* (Spek, 2009 ▶); software used to prepare material for publication: *SHELXL97*.

## Supplementary Material

Crystal structure: contains datablocks global, I. DOI: 10.1107/S1600536809010526/at2748sup1.cif

Structure factors: contains datablocks I. DOI: 10.1107/S1600536809010526/at2748Isup2.hkl

Additional supplementary materials:  crystallographic information; 3D view; checkCIF report

## Figures and Tables

**Fig. 1.**
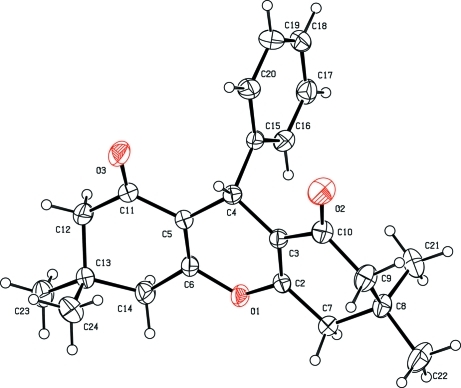
The molecular structure of the title compound, showing 30% probability displacement ellipsoids and the atom-numbering scheme.

## supplementary crystallographic information

### Comment

Xanthenes are an important class of organic compounds that received considerable attention from many pharmaceuticals and organic chemists, actually because of the broad spectrum of their biological and pharmaceutical properties such as agricultural bactericide effects (Hideo, 1981), photodynamic therapy, anti-inflammatory activities (Poupelin *et al.*, 1978) and antiviral effects (Lambert *et al.*, 1997). Considering the importance of the title compound (I), we report here the crystal structure of it.

In the molecule of (I), (Fig. 1), rings A(C14—C6), B(O1/C6/C5/C4/C3/C2) and C(C2—C7) are not planar, having total puckering amplitudes, Q_T_, of 0.443 (2), 0.202 (2) and 0.449 (2) Å, respectively. They adopt envelope [Φ = 12.2 (3) and θ = 130.5 (2) °], boat [Φ = 351.8 (5) and θ = 102.5 (5) °] and envelope [Φ = 45.2 (4) and θ = 125.6 (2) °] conformations (Cremer & Pople, 1975). In rings A and C, atoms C13 and C8 are displaced by 0.609 (1) and 0.616 (1) Å from the plane of the other ring atoms, respectively. Ring D(C15—C20) is, of course, planar.

The crystal structure is stabilized by van der Waals interactions.

### Experimental

A mixture of benzaldehyde (10 mmol), 5, 5-dimethyl-1,3-cyclohexanedione (2. 20 mmol) were mixed along with 20 ml of ethanol, to that ammonium acetate (10 mmol) was added and refluxed on waterbath for about 1 h. The progress of the reaction was monitored by TLC. After conforming that the reaction got completed, the reaction mixture was allowed to cool to room temperature and left aside for a day. Yellow colour solid crystals were started growing from the mother liquor. It was filtered and washed with diethyl ether to ensure pure crystals [yield: 91%, m.p. 478–480 K].

### Refinement

The H atoms were placed in calculated positions and allowed to ride on their carrier atoms with C—H = 0.93–0.98 Å and with *U*_iso_(H) = 1.2*U*_eq_(C) for CH, CH_2_ and *U*_iso_(H) = 1.5*U*_eq_(C) for CH_3_ groups.

### Crystal data

**Table:**

C_23_H_26_O_3_	*F*(000) = 752
*M**_r_* = 350.44	*D*_x_ = 1.195 Mg m^−^^3^
Monoclinic, *P*2_1_/*c*	Mo *K*α radiation, λ = 0.71073 Å
Hall symbol: -P 2ybc	Cell parameters from 2500 reflections
*a* = 6.0562 (5) Å	θ = 2–27°
*b* = 19.7680 (18) Å	µ = 0.08 mm^−^^1^
*c* = 16.4325 (13) Å	*T* = 293 K
β = 97.924 (3)°	Needle, colourless
*V* = 1948.5 (3) Å^3^	0.17 × 0.15 × 0.11 mm
*Z* = 4	

### Data collection

**Table:**

Bruker SMART APEX CCD diffractometer	4284 independent reflections
Radiation source: fine-focus sealed tube	2825 reflections with *I* > 2σ(*I*)
graphite	*R*_int_ = 0.035
ω scans	θ_max_ = 27.1°, θ_min_ = 1.6°
Absorption correction: multi-scan (*SADABS*; Bruker, 1998)	*h* = −5→7
*T*_min_ = 0.987, *T*_max_ = 0.992	*k* = −25→25
11861 measured reflections	*l* = −21→12

### Refinement

**Table:**

Refinement on *F*^2^	Primary atom site location: structure-invariant direct methods
Least-squares matrix: full	Secondary atom site location: difference Fourier map
*R*[*F*^2^ > 2σ(*F*^2^)] = 0.050	Hydrogen site location: inferred from neighbouring sites
*wR*(*F*^2^) = 0.138	H-atom parameters constrained
*S* = 1.03	*w* = 1/[σ^2^(*F*_o_^2^) + (0.0661*P*)^2^ + 0.1411*P*] where *P* = (*F*_o_^2^ + 2*F*_c_^2^)/3
4284 reflections	(Δ/σ)_max_ < 0.001
239 parameters	Δρ_max_ = 0.17 e Å^−^^3^
0 restraints	Δρ_min_ = −0.20 e Å^−^^3^

### Special details

**Table:**

Geometry. All e.s.d.'s (except the e.s.d. in the dihedral angle between two l.s. planes) are estimated using the full covariance matrix. The cell e.s.d.'s are taken into account individually in the estimation of e.s.d.'s in distances, angles and torsion angles; correlations between e.s.d.'s in cell parameters are only used when they are defined by crystal symmetry. An approximate (isotropic) treatment of cell e.s.d.'s is used for estimating e.s.d.'s involving l.s. planes.
Refinement. Refinement of *F*^2^ against ALL reflections. The weighted *R*-factor *wR* and goodness of fit *S* are based on *F*^2^, conventional *R*-factors *R* are based on *F*, with *F* set to zero for negative *F*^2^. The threshold expression of *F*^2^ > σ(*F*^2^) is used only for calculating *R*-factors(gt) *etc*. and is not relevant to the choice of reflections for refinement. *R*-factors based on *F*^2^ are statistically about twice as large as those based on *F*, and *R*- factors based on ALL data will be even larger.

### Fractional atomic coordinates and isotropic or equivalent isotropic displacement parameters (Å^2^)

**Table:**

	*x*	*y*	*z*	*U*_iso_*/*U*_eq_	
C2	0.2342 (3)	0.26716 (7)	0.39187 (9)	0.0364 (4)	
C3	0.0380 (2)	0.27207 (7)	0.34340 (9)	0.0353 (3)	
C4	−0.0306 (3)	0.22377 (7)	0.27309 (9)	0.0363 (4)	
H4	−0.1866	0.2110	0.2741	0.044*	
C5	0.1110 (3)	0.16067 (8)	0.28674 (9)	0.0379 (4)	
C6	0.3004 (3)	0.15872 (7)	0.33973 (9)	0.0390 (4)	
C7	0.3232 (3)	0.31533 (8)	0.45793 (9)	0.0424 (4)	
H7A	0.4811	0.3220	0.4562	0.051*	
H7B	0.3068	0.2956	0.5108	0.051*	
C8	0.2064 (3)	0.38413 (8)	0.45065 (10)	0.0450 (4)	
C9	−0.0457 (3)	0.37197 (10)	0.43150 (11)	0.0544 (5)	
H9A	−0.0970	0.3516	0.4792	0.065*	
H9B	−0.1199	0.4153	0.4221	0.065*	
C10	−0.1139 (3)	0.32744 (9)	0.35821 (10)	0.0447 (4)	
C11	0.0384 (3)	0.09875 (8)	0.24133 (10)	0.0479 (4)	
C12	0.1983 (4)	0.03983 (9)	0.24839 (11)	0.0607 (5)	
H12A	0.2987	0.0452	0.2077	0.073*	
H12B	0.1141	−0.0014	0.2355	0.073*	
C13	0.3369 (3)	0.03208 (8)	0.33293 (10)	0.0497 (4)	
C14	0.4530 (3)	0.09951 (8)	0.35552 (11)	0.0504 (4)	
H14A	0.5142	0.0988	0.4133	0.060*	
H14B	0.5761	0.1048	0.3240	0.060*	
C15	−0.0128 (3)	0.25771 (7)	0.19070 (9)	0.0360 (4)	
C16	0.1833 (3)	0.28868 (9)	0.17640 (10)	0.0457 (4)	
H16	0.3073	0.2874	0.2167	0.055*	
C17	0.1970 (3)	0.32159 (9)	0.10281 (11)	0.0534 (5)	
H17	0.3297	0.3421	0.0939	0.064*	
C18	0.0143 (4)	0.32390 (9)	0.04283 (11)	0.0562 (5)	
H18	0.0228	0.3465	−0.0063	0.067*	
C19	−0.1807 (3)	0.29273 (10)	0.05584 (11)	0.0601 (5)	
H19	−0.3039	0.2939	0.0153	0.072*	
C20	−0.1939 (3)	0.25964 (9)	0.12930 (10)	0.0495 (4)	
H20	−0.3261	0.2384	0.1375	0.059*	
C21	0.2861 (3)	0.42570 (9)	0.38171 (13)	0.0613 (5)	
H21A	0.2137	0.4690	0.3783	0.092*	
H21B	0.2502	0.4022	0.3305	0.092*	
H21C	0.4446	0.4319	0.3931	0.092*	
C22	0.2621 (4)	0.42158 (12)	0.53235 (13)	0.0775 (7)	
H22A	0.4210	0.4241	0.5467	0.116*	
H22B	0.1990	0.3977	0.5745	0.116*	
H22C	0.2012	0.4665	0.5271	0.116*	
C23	0.5131 (4)	−0.02346 (10)	0.32996 (14)	0.0785 (7)	
H23A	0.4405	−0.0654	0.3136	0.118*	
H23B	0.5982	−0.0285	0.3834	0.118*	
H23C	0.6106	−0.0111	0.2910	0.118*	
C24	0.1837 (4)	0.01311 (11)	0.39644 (13)	0.0756 (6)	
H24A	0.1096	−0.0288	0.3808	0.113*	
H24B	0.0748	0.0481	0.3987	0.113*	
H24C	0.2710	0.0082	0.4495	0.113*	
O1	0.37769 (18)	0.21342 (5)	0.38742 (7)	0.0448 (3)	
O2	−0.2918 (2)	0.33452 (7)	0.31414 (8)	0.0672 (4)	
O3	−0.1434 (2)	0.09604 (6)	0.19873 (9)	0.0699 (4)	

### Atomic displacement parameters (Å^2^)

**Table:**

	*U*^11^	*U*^22^	*U*^33^	*U*^12^	*U*^13^	*U*^23^
C2	0.0412 (9)	0.0298 (8)	0.0381 (8)	0.0020 (7)	0.0052 (7)	0.0017 (6)
C3	0.0360 (8)	0.0329 (8)	0.0367 (8)	0.0003 (7)	0.0043 (6)	−0.0007 (6)
C4	0.0323 (8)	0.0352 (8)	0.0397 (8)	−0.0005 (7)	−0.0007 (6)	−0.0004 (6)
C5	0.0453 (9)	0.0316 (8)	0.0358 (8)	0.0009 (7)	0.0018 (7)	0.0005 (6)
C6	0.0431 (9)	0.0298 (8)	0.0428 (8)	0.0002 (7)	0.0018 (7)	−0.0019 (7)
C7	0.0490 (10)	0.0378 (9)	0.0388 (8)	−0.0017 (8)	−0.0003 (7)	−0.0023 (7)
C8	0.0460 (10)	0.0368 (9)	0.0524 (10)	0.0014 (8)	0.0074 (8)	−0.0094 (7)
C9	0.0492 (11)	0.0530 (11)	0.0636 (11)	0.0044 (9)	0.0172 (9)	−0.0163 (9)
C10	0.0398 (9)	0.0451 (10)	0.0501 (9)	0.0030 (8)	0.0096 (8)	−0.0026 (8)
C11	0.0638 (12)	0.0382 (9)	0.0386 (9)	−0.0004 (8)	−0.0042 (8)	−0.0007 (7)
C12	0.0880 (15)	0.0391 (10)	0.0497 (10)	0.0125 (10)	−0.0097 (10)	−0.0101 (8)
C13	0.0689 (12)	0.0319 (9)	0.0455 (9)	0.0069 (8)	−0.0020 (8)	−0.0009 (7)
C14	0.0518 (10)	0.0384 (9)	0.0579 (10)	0.0084 (8)	−0.0028 (8)	−0.0019 (8)
C15	0.0395 (8)	0.0305 (8)	0.0363 (8)	0.0027 (7)	−0.0006 (6)	−0.0030 (6)
C16	0.0452 (10)	0.0467 (10)	0.0440 (9)	−0.0038 (8)	0.0018 (7)	−0.0049 (8)
C17	0.0653 (12)	0.0435 (10)	0.0540 (10)	−0.0106 (9)	0.0178 (9)	−0.0038 (8)
C18	0.0869 (15)	0.0412 (10)	0.0406 (9)	0.0031 (10)	0.0093 (9)	0.0046 (8)
C19	0.0665 (13)	0.0629 (12)	0.0457 (10)	0.0049 (11)	−0.0113 (9)	0.0072 (9)
C20	0.0447 (10)	0.0511 (11)	0.0491 (10)	−0.0035 (8)	−0.0065 (8)	0.0037 (8)
C21	0.0562 (12)	0.0397 (10)	0.0881 (14)	0.0017 (9)	0.0101 (10)	0.0126 (10)
C22	0.0799 (16)	0.0678 (14)	0.0827 (15)	0.0012 (12)	0.0036 (12)	−0.0377 (12)
C23	0.1014 (17)	0.0442 (11)	0.0823 (15)	0.0268 (12)	−0.0138 (13)	−0.0109 (10)
C24	0.0986 (17)	0.0577 (13)	0.0692 (13)	−0.0073 (12)	0.0070 (12)	0.0173 (10)
O1	0.0426 (6)	0.0322 (6)	0.0551 (7)	0.0053 (5)	−0.0092 (5)	−0.0062 (5)
O2	0.0462 (8)	0.0734 (10)	0.0784 (9)	0.0206 (7)	−0.0042 (7)	−0.0149 (7)
O3	0.0744 (10)	0.0486 (8)	0.0766 (9)	−0.0028 (7)	−0.0251 (8)	−0.0129 (7)

### Geometric parameters (Å, °)

**Table:**

C2—C3	1.340 (2)	C13—C24	1.536 (3)
C2—O1	1.3809 (17)	C13—C23	1.536 (3)
C2—C7	1.488 (2)	C14—H14A	0.9700
C3—C10	1.471 (2)	C14—H14B	0.9700
C3—C4	1.512 (2)	C15—C20	1.384 (2)
C4—C5	1.513 (2)	C15—C16	1.385 (2)
C4—C15	1.528 (2)	C16—C17	1.386 (2)
C4—H4	0.9800	C16—H16	0.9300
C5—C6	1.341 (2)	C17—C18	1.377 (3)
C5—C11	1.469 (2)	C17—H17	0.9300
C6—O1	1.3788 (18)	C18—C19	1.375 (3)
C6—C14	1.492 (2)	C18—H18	0.9300
C7—C8	1.530 (2)	C19—C20	1.385 (2)
C7—H7A	0.9700	C19—H19	0.9300
C7—H7B	0.9700	C20—H20	0.9300
C8—C22	1.529 (2)	C21—H21A	0.9600
C8—C21	1.531 (2)	C21—H21B	0.9600
C8—C9	1.534 (2)	C21—H21C	0.9600
C9—C10	1.503 (2)	C22—H22A	0.9600
C9—H9A	0.9700	C22—H22B	0.9600
C9—H9B	0.9700	C22—H22C	0.9600
C10—O2	1.221 (2)	C23—H23A	0.9600
C11—O3	1.222 (2)	C23—H23B	0.9600
C11—C12	1.509 (2)	C23—H23C	0.9600
C12—C13	1.529 (2)	C24—H24A	0.9600
C12—H12A	0.9700	C24—H24B	0.9600
C12—H12B	0.9700	C24—H24C	0.9600
C13—C14	1.529 (2)		
			
C3—C2—O1	122.50 (13)	C14—C13—C23	109.43 (16)
C3—C2—C7	126.13 (14)	C24—C13—C23	109.54 (17)
O1—C2—C7	111.33 (13)	C6—C14—C13	112.88 (14)
C2—C3—C10	118.61 (14)	C6—C14—H14A	109.0
C2—C3—C4	122.44 (13)	C13—C14—H14A	109.0
C10—C3—C4	118.94 (13)	C6—C14—H14B	109.0
C3—C4—C5	108.46 (12)	C13—C14—H14B	109.0
C3—C4—C15	110.74 (12)	H14A—C14—H14B	107.8
C5—C4—C15	112.66 (12)	C20—C15—C16	118.41 (14)
C3—C4—H4	108.3	C20—C15—C4	120.80 (14)
C5—C4—H4	108.3	C16—C15—C4	120.77 (13)
C15—C4—H4	108.3	C15—C16—C17	120.76 (16)
C6—C5—C11	118.38 (14)	C15—C16—H16	119.6
C6—C5—C4	122.44 (13)	C17—C16—H16	119.6
C11—C5—C4	119.17 (14)	C18—C17—C16	120.09 (17)
C5—C6—O1	122.64 (13)	C18—C17—H17	120.0
C5—C6—C14	126.09 (14)	C16—C17—H17	120.0
O1—C6—C14	111.27 (13)	C19—C18—C17	119.77 (16)
C2—C7—C8	113.26 (13)	C19—C18—H18	120.1
C2—C7—H7A	108.9	C17—C18—H18	120.1
C8—C7—H7A	108.9	C18—C19—C20	120.08 (17)
C2—C7—H7B	108.9	C18—C19—H19	120.0
C8—C7—H7B	108.9	C20—C19—H19	120.0
H7A—C7—H7B	107.7	C15—C20—C19	120.88 (17)
C22—C8—C7	108.54 (15)	C15—C20—H20	119.6
C22—C8—C21	109.63 (16)	C19—C20—H20	119.6
C7—C8—C21	110.20 (14)	C8—C21—H21A	109.5
C22—C8—C9	110.41 (15)	C8—C21—H21B	109.5
C7—C8—C9	108.23 (14)	H21A—C21—H21B	109.5
C21—C8—C9	109.80 (15)	C8—C21—H21C	109.5
C10—C9—C8	114.33 (14)	H21A—C21—H21C	109.5
C10—C9—H9A	108.7	H21B—C21—H21C	109.5
C8—C9—H9A	108.7	C8—C22—H22A	109.5
C10—C9—H9B	108.7	C8—C22—H22B	109.5
C8—C9—H9B	108.7	H22A—C22—H22B	109.5
H9A—C9—H9B	107.6	C8—C22—H22C	109.5
O2—C10—C3	120.52 (15)	H22A—C22—H22C	109.5
O2—C10—C9	122.07 (15)	H22B—C22—H22C	109.5
C3—C10—C9	117.34 (15)	C13—C23—H23A	109.5
O3—C11—C5	120.72 (16)	C13—C23—H23B	109.5
O3—C11—C12	121.90 (15)	H23A—C23—H23B	109.5
C5—C11—C12	117.37 (15)	C13—C23—H23C	109.5
C11—C12—C13	114.43 (14)	H23A—C23—H23C	109.5
C11—C12—H12A	108.7	H23B—C23—H23C	109.5
C13—C12—H12A	108.7	C13—C24—H24A	109.5
C11—C12—H12B	108.7	C13—C24—H24B	109.5
C13—C12—H12B	108.7	H24A—C24—H24B	109.5
H12A—C12—H12B	107.6	C13—C24—H24C	109.5
C12—C13—C14	108.04 (14)	H24A—C24—H24C	109.5
C12—C13—C24	109.55 (17)	H24B—C24—H24C	109.5
C14—C13—C24	110.41 (15)	C6—O1—C2	117.81 (11)
C12—C13—C23	109.85 (14)		
			
O1—C2—C3—C10	173.83 (13)	C6—C5—C11—O3	172.63 (16)
C7—C2—C3—C10	−3.8 (2)	C4—C5—C11—O3	−6.6 (2)
O1—C2—C3—C4	−7.5 (2)	C6—C5—C11—C12	−8.5 (2)
C7—C2—C3—C4	174.82 (14)	C4—C5—C11—C12	172.31 (15)
C2—C3—C4—C5	19.19 (19)	O3—C11—C12—C13	−145.42 (19)
C10—C3—C4—C5	−162.15 (13)	C5—C11—C12—C13	35.7 (2)
C2—C3—C4—C15	−104.92 (16)	C11—C12—C13—C14	−53.6 (2)
C10—C3—C4—C15	73.74 (17)	C11—C12—C13—C24	66.7 (2)
C3—C4—C5—C6	−16.4 (2)	C11—C12—C13—C23	−172.93 (18)
C15—C4—C5—C6	106.60 (16)	C5—C6—C14—C13	−23.1 (2)
C3—C4—C5—C11	162.83 (13)	O1—C6—C14—C13	156.85 (14)
C15—C4—C5—C11	−74.21 (18)	C12—C13—C14—C6	46.2 (2)
C11—C5—C6—O1	−177.46 (14)	C24—C13—C14—C6	−73.61 (19)
C4—C5—C6—O1	1.7 (2)	C23—C13—C14—C6	165.75 (16)
C11—C5—C6—C14	2.4 (2)	C3—C4—C15—C20	−125.34 (15)
C4—C5—C6—C14	−178.36 (15)	C5—C4—C15—C20	112.98 (16)
C3—C2—C7—C8	−17.9 (2)	C3—C4—C15—C16	53.10 (19)
O1—C2—C7—C8	164.25 (13)	C5—C4—C15—C16	−68.58 (18)
C2—C7—C8—C22	164.25 (16)	C20—C15—C16—C17	0.9 (2)
C2—C7—C8—C21	−75.68 (18)	C4—C15—C16—C17	−177.63 (15)
C2—C7—C8—C9	44.40 (18)	C15—C16—C17—C18	0.1 (3)
C22—C8—C9—C10	−172.24 (16)	C16—C17—C18—C19	−0.8 (3)
C7—C8—C9—C10	−53.6 (2)	C17—C18—C19—C20	0.6 (3)
C21—C8—C9—C10	66.8 (2)	C16—C15—C20—C19	−1.1 (2)
C2—C3—C10—O2	178.27 (16)	C4—C15—C20—C19	177.38 (16)
C4—C3—C10—O2	−0.4 (2)	C18—C19—C20—C15	0.4 (3)
C2—C3—C10—C9	−4.7 (2)	C5—C6—O1—C2	12.5 (2)
C4—C3—C10—C9	176.63 (14)	C14—C6—O1—C2	−167.45 (13)
C8—C9—C10—O2	−148.28 (17)	C3—C2—O1—C6	−9.5 (2)
C8—C9—C10—C3	34.7 (2)	C7—C2—O1—C6	168.44 (13)
